# Delayed gastric emptying after pancreatoduodenectomy: comparison between invaginated pancreatogastrostomy and pancreatojejunostomy

**DOI:** 10.1186/s12893-020-00707-w

**Published:** 2020-04-03

**Authors:** S. Hayama, N. Senmaru, S. Hirano

**Affiliations:** 1 Department of Surgery, Steel Memorial Muroran Hospital, 1-45 Chiribetucyo, Muroran, Hokkaido 050-0076 Japan; 2 Department of Gastroenterological Surgery II, Hokkaido University Graduate School of Medicine, N−15, W−7, Kita-ku, Sapporo, Japan

**Keywords:** Delayed gastric emptying, Pancreatoduodenectomy, Intra-abdominal complication, Pancreatic fistula, Pancreatic reconstruction

## Abstract

**Background:**

The association between delayed gastric emptying (DGE) after pancreatoduodenectomy (PD) and pancreatic reconstruction technique remain unclear. The aim of this study is to investigate whether the occurrence of DGE differs between pancreaticojejunostomy (PJ) and pancreaticogastrostomy (PG).

**Methods:**

A total of 83 patients who underwent subtotal stomach-preserving pancreatoduodenectomy was retrospectively analyzed, and the factors associated with clinically relevant DGE were explored. These patients were divided into a PG group and a PJ group according to the pancreatic reconstruction. DGE occurrence and its association with intra-abdominal complications was compared between the two types of pancreatic reconstruction.

**Results:**

The overall incidence of DGE was 27.7%. Intra-abdominal complications including pancreatic fistula were strongly associated with DGE. As to the pancreatic reconstruction, DGE developed more frequently in the PG than in the PJ. In addition, DGE with intra-abdominal complications tended to be more frequent in PG, despite the fact that intra-abdominal complications occurred at a similar frequency in both groups.

**Conclusions:**

Intra-abdominal complications were strongly associated with DGE. As to the pancreatic reconstruction, DGE developed more frequently in the PG than in the PJ. We speculate that intra-abdominal complications affected patients with PG more and resulted in frequent occurrence of DGE.

## Background

The mortality rate after pancreatoduodenectomy (PD) is declining to a very low level in specialized centers. However, the postoperative morbidity rate remains high (approximately 50%) [1, 2], and delayed gastric emptying (DGE) is the most predominant morbidity, with an incidence varying from 19 to 57% [2, 3]. Although not life-threatening, DGE is associated with a decreased quality of life, increased postoperative length of stay, and increased healthcare costs.

Because of the variation in the definition of DGE, it has not been possible to compare studies of DGE [4]. To resolve this issue, the International Study Group of Pancreatic Surgery (ISGPS) developed an objective and generally applicable definition in 2006 based on two clinical parameters [4]: number of days that nasogastric drainage is required, and the number of days until solid food is tolerated. As a result, reports about DGE defined according to this generalized definition have been accumulating [3, 5, 6]. Regarding the pathogenesis of DGE, denervation of the antropyloric region, pyloric and antral ischemia, and decreased levels of motilin have been suggested [6, 7]. Furthermore, strong associations with other postoperative complications, especially intra-abdominal complications (IACs) such as pancreatic fistula (PF) and intra-abdominal abscesses (IAAs), have been reported [4–7]. Considering the association between DGE and IACs, the procedure for remnant pancreatic reconstruction would likely be a crucial factor related to DGE occurrence, because its disruption would result in various kinds of IACs. To minimize the risk of pancreatic anastomosis failure, pancreaticogastrostomy (PG) has been adopted instead of pancreaticojejunostomy (PJ) by several surgeons [8, 9]. However, there has been no definite evidence showing increased safety with PG [10]. Although several resent meta-analyses reported DGE occurrence was comparable between PJ and PG, there seemed to be numerous variations in the performance of PG in these meta-analyses [1, 9, 11, 12]. Therefore, there is still room for further consideration of the issue. In the present study, both PJ and PG (invagination procedure) were performed uniformly in all patients. Thus, the aim of the present study was to compare the occurrence of clinically relevant DGE and its association with IACs between these two different pancreatic reconstruction techniques.

## Methods

Of a consecutive series of 93 patients undergoing elective PD from May 2002 to March 2012 in the Steel Memorial Muroran Hospital, a retrospective review of 83 patients who recovered and were discharged from hospital was carried out. The following 10 patients were excluded from this analysis: two patients who died from postoperative complications (pneumonia and acute myocardial Infarction) within 7th postoperative day, one patient died from rapid growth of liver metastasis during hospitalization who has never taken any solid food nor water due to severe general fatigue, and seven patients whose clinical records were not available after the retention period has lapsed. DGE analysis according to the definition was impossible in these 10 patients. All of the patients underwent subtotal stomach-preserving pancreatoduodenectomy (SSPPD). Prior to 2008, PG was used exclusively for remnant pancreatic reconstruction; in 2009, PJ was adopted for reconstruction instead of PG. The reason was that DGE occurrence as well as pancreatic duct obstruction were relatively frequent in PG. All operations were performed by experienced surgeons.

### Descriptions of the operative procedures

The procedure for the resection at the time of SSPPD was the same for the PG and PJ groups; the gallbladder, distal common bile duct, head of the pancreas, duodenum, and about 10 cm of the proximal jejunum were removed. The antrum was resected 3 cm oral to the pyloric ring. The reconstruction procedures for the PG and PJ groups were as follows.

### PG group

PG was performed with an invagination technique, hand-sewn with absorbable monofilament sutures, on the posterior wall of the gastric body, and a short internal stent was used. The proximal jejunum was brought through the transverse mesocolon by the retrocolic route, end-to-side choledochojejunostomy was performed using interrupted absorbable monofilament sutures with or without tube drainage, according to duct size. Further downstream, after a loop gastrojejunostomy, anastomosis between afferent and efferent loops of jejunum (Braun procedure) was performed.

### PJ group

The jejunal loop was brought in the same manner as in the PG group. PJ with end-to-side technique was performed between the pancreatic duct and the jejunal mucosa using 8 to 12 interrupted absorbable monofilament sutures according to duct size. A pancreatic duct tube was routinely placed in the main pancreatic duct. Further downstream, end-to-side choledochojejunostomy, a loop gastrojejunostomy and anastomosis between afferent and efferent loops of jejunum were performed in a similar manner to PG group. Closed suction was placed posterior to the biliary and pancreatic anastomosis in both groups.

### Perioperative management

There were no differences in the perioperative management between the two groups.

In both groups, the gastric suction using a nasogastric tube was removed when the daily discharge was less than 500 mL, usually on the first postoperative day. Solid oral intake was initiated 4 days after operation and the patients proceeded to a regular diet within about 14 days.

### Classification of delayed gastric emptying (DGE) and pancreatic fistula

The severity of DGE was determined according to the ISGPS classification scheme [4] in both group. Grades B and C DGE in this classification were defined as clinically relevant DGE. Clinically relevant DGE was further divided into two types of DGE (primary DGE and secondary DGE), according to the presence of IACs. Patency of the duodenojejunostomy to exclude a mechanical cause of abnormal gastric emptying was confirmed by an upper gastrointestinal series.

The grade of PF was determined according to the ISGPS classification scheme [13], and grades B and C PF in this classification were defined as clinically relevant PF.

### Statistical analysis

Statflex version 6 software (Artech Co., Ltd., Osaka, Japan) was used for all statistical analyses. The χ^2^ and Fisher’s exact test were used for categorical variables, and Student’s *t-*test was used for continuous variables. Values of *p* < 0.05 were considered significant.

## Results

The characteristics of the 83 participants are shown in Table 1. The median age at the time of surgery was 66 years (range, 50–82 years), and 34.9% of all patients were women. For the remnant pancreatic reconstruction, PG was used in 37 patients (44.6%) prior to 2008, whereas PJ was used for 46 patients (55.4%) after 2009. The overall mortality rate was 2.4% (*n* = 2). Morbidity was noted in 46 patients (55.4%), and IACs were seen in 37 (44.6%). The IACs in this study included PF, primary IAA, chylorrhea, postoperative hemorrhage, enterocolitis, liver abscess, pancreatitis, and gastric ulcer bleeding. The most frequent complication was DGE; 23 patients (27.7%) developed clinically relevant DGE, classified as grades B (*n* = 4) and C (*n* = 19). Of the 23 clinically relevant DGE patients, 20 patients (87.0%) had DGE with IACs, and 3 patients (13.0%) had DGE without IACs. The nasogastric tube was needed beyond the 2th postoperative day in 11 patients (47.8%), and reinsertion was noted in 9 patients (39.1%) of the 23 clinically relevant DGE patients. The median postoperative hospital stay was 32 days (range, 12–146 days).

**Table 1:**
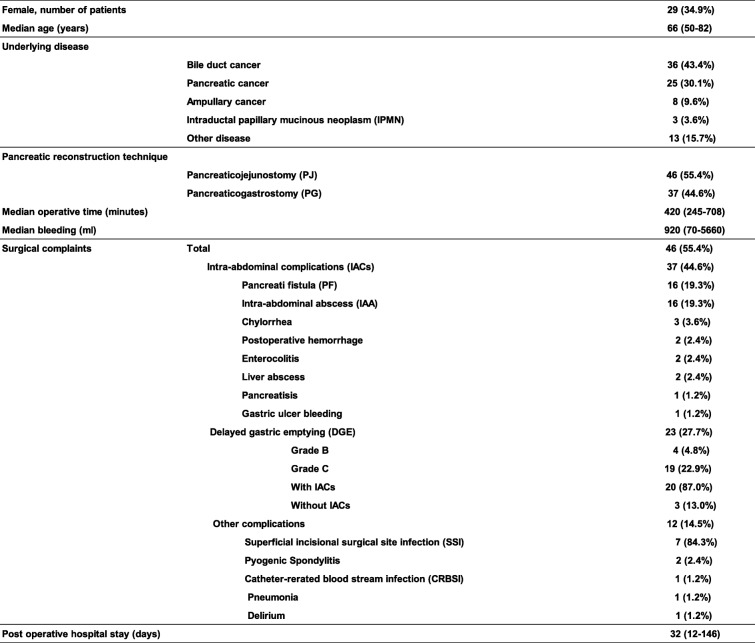
Summary of the 83 Patients Characteristics of the Study Population

The baseline characteristics of PJ (*n* = 46) and PG (*n* = 37) cohorts are summarized in Table 2. There were no significant associations with median age, sex ratio, median body mass index (BMI) and preoperative cholangitis/biliary drainage. As to the underlying disease, pancreatic cancer was more frequent in PG group.

**Table 2:**
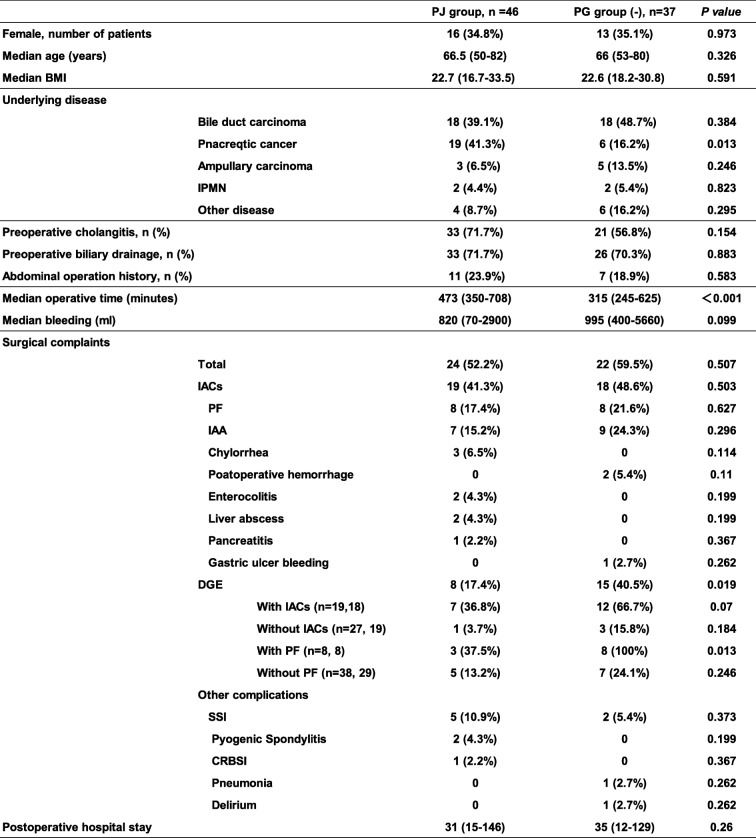
Comparison of patients’ characteristics between the PJ and PG groups

Postoperative features were also compared between PJ and PG cohorts in Table 2. Operative time was significantly longer whereas intraoperative blood loss tend to be less in the PJ group compared with PG group (*p <* 0.001 and 0.99, respectivly). The surgical complaints were generally similar between the two types of reconstruction, except for DGE occurrence, which occurred more frequently in the PG group than in the PJ group (40.5% vs. 17.4%, respectively, *p* = 0.019). Remarkably, delayed gastric emptying with intra-abdominal complications tended to be more frequent in pancreaticogastrostomy, despite the fact that intra-abdominal complications occurred at a similar frequency in both groups (36.8% vs. 66.7%, respectively, *p* = 0.07). In particular, DGE with PF was more frequent in PG (37.5% vs. 100%, respectively, *p* = 0.013).

Multivariate analysis using logistic regression was shown in Table 3, identified thee variables as independently associated with the occurrence of DGE: pancreatic fistula, intra-abdominal abscess and pancreatic reconstruction technique.

**Table 3:**
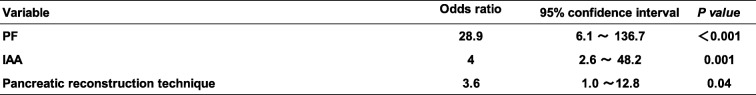
Factors associated with delayed gastric emptying in multivaliate analysis

## Discussion

Recent investigations have reported that DGE should be considered a warning of IACs, such as PF or IAA [6, 8, 14–16]. In fact, DGE is most often secondary to the presence of IACs (secondary DGE), and very seldom a primary event (primary DGE) [6, 7, 17]. Similarly, in the present study, most DGE patients (*n* = 20, 87%) had IACs, which confirmed this association (*p* < 0.001), whereas primary DGE was rare (*n* = 3, 13%). As to surgical procedure, pylorus-preserving pancreaticoduodenectomy (PPPD) [18] and a postcolic route for the GE reconstruction [19] have been reported as typical risk factors for DGE. In our hospital, resection of pylorus ring with preservation of nearly the entire stomach (pylorus-resecting pancreaticoduodenectomy; PrPD), antecolic route have been adopted for GE reconstruction. Similarly, Kawai et al. reported that PrPD in which 95% of stomach was preserved resulted in very low incidence of DGE (18). On the other hand, the reconstruction method of the pancreatic stump is closely associated with IACs, which have been frequently reported as risk factors for DGE [3, 6]. Considering this relationship, a close association between the procedure of pancreatic reconstruction and DGE occurrence is expected. Although several resent meta-analyses reported DGE occurrence was comparable between PJ and PG, there seemed to be numerous variations in the performance of PG in these meta-analyses [1, 9, 11, 12]. In the present study, both PJ and PG (invagination procedure) were performed uniformly in all patients and thus any reconstruction bias that might have influenced DGE was almost eliminated. Moreover, PJ was exclusively adopted for pancreatic reconstruction in SSPPD instead of PG since 2009. There was no bias in the selection of patients between PG and PJ even though the present study was retrospective, which adds to the validity of the present analysis. As expected, and this was the most striking result, DGE occurred more frequently in the PG group.

The frequent occurrence of DGE in PG would suggest that the surgical procedure of PG itself affected DGE occurrence. It is possible that the PG resulted in fixation of the subtotal stomach to the posterior wall, thereby disturbing gastric peristalsis.

In particular, PG in the present study was performed with an invagination technique, which contained more anatomical destruction than PG with duct-to-mucosa anastomosis and could lead to more severe disturbance of gastric peristalsis, in pancreatic fistula or other intraabdominal complications.

Next, as to DGE with IACs, safer pancreatic reconstruction with fewer IACs should decrease this type of DGE. Some surgeons prefer PG to PJ for patients at high risk of PF because some observational clinical studies (OCSs) reported a lower incidence of PF with PG [8, 9]. However, these reports showed high heterogeneity, and, moreover, no high-quality, randomized, controlled trials (RCTs) have yet to provide adequate evidence of greater safety with PG than with PJ [10]. Similarly, in the present study, there were no significant differences in the frequencies of PF and IACs between the PG and PJ groups, but DGE with IACs occurred more frequently in the PG group. As suspected, the present data suggested that the PG group was more vulnerable than the PJ group to the impact of DGE occurring by IACs. Especially with regard to PF, a strong association with DGE was shown in the PG group, but not in the PJ group. In the PJ group, 3 of 8 patients (37.5%) with PF had DGE, whereas all PG patients with PF were affected by DGE (*P* = 0.007). It is probable that, in patients with PJ, the increasing distance from the pancreatic anastomosis would reduce gastric paresis due to PF or peripancreatic inflammation.

Although pancreatic anastomosis should not be chosen based on prevention of DGE alone, PG appeared to have a tendency to induce DGE, and thereby resulted in patient frustration, the need for nutritional support, and prolonged hospital stay. When PG is adopted, surgeons should take care to prevent disturbance of gastric peristalsis, including the anastomotic procedure, by omitting the incision on the anterior gastric wall, or choosing a vertical incision instead of a horizontal incision [20].

Although the ISGPS criteria have enabled comparisons of DGE between investigators using the general definition, interpretation of DGE is sometimes confusing. For example, patients with IACs, including chylorrhea, postoperative hemorrhage, enterocolitis, and gastric ulcer bleeding, may require withdrawal of oral intake despite lacking gastroparesis. DGE was originally described as gastroparesis following PPPD [21]; therefore, in our opinion, these patients should not be categorized as having DGE, whereas some investigators might have interpreted such cases as having DGE. This confusion occurred because the ISGPS criteria did not mention the presence or absence of co-existing complications, exclusion criteria, and the method for evaluating gastroparesis, although the criteria are simple, objective, and clearly measurable. Refinement of the definition is warranted for further analysis of the etiology of DGE.

The limitation of the current study is that the number of patients was small and there was a slight difference in surgical devises or drugs between PJ and PG cohorts depending on the period. Accumulation of additional cases with minimal variations is needed to definitively characterize the risk for DGE between PJ and PG cohorts in the future.

In conclusion, the occurrence of DGE and its association with IACs was compared between different pancreatic reconstruction techniques. Intra-abdominal complications including PF were strongly associated with DGE. As to the pancreatic reconstruction, DGE developed more frequently in the PG than in the PJ. We speculate that PG itself predisposed patients to DGE by the fixation to the posterior wall and intra-abdominal complications affected patients with PG more, these resulted in frequent occurrence of DGE in PG.

## Data Availability

All data and materials are contained within the manuscript.

## Abbreviations

DGE: Delayed gastric emptying
IAA: Intra-abdominal abscess
IAC: Intra-abdominal complication
ISGPS: International Study Group of Pancreatic Surgery
PD: Pancreatoduodenectomy
PF: Pancreatic fistula
PG: Pancreaticogastrostomy
PJ: Pancreaticojejunostomy
PPPD: Pylorus-preserving pancreaticoduodenectomy
PrPD: Pylorus-resecting pancreaticoduodenectomy
SSPPD: Subtotal stomach-preserving pancreatoduodenectomy
